# Hybrid PET/fMRI reveals interactive effects of T2DM and MCI on cerebral glucose metabolism and ALFF

**DOI:** 10.3389/fendo.2026.1778600

**Published:** 2026-03-20

**Authors:** Zheng Li, Jiao Liu, Cuihua Zhao, Zhi Zou, Bo Dai, Zhonglin Li

**Affiliations:** 1 Department of Radiology, Henan Provincial People’s Hospital & People’s Hospital of Zhengzhou University, Zhengzhou, China; 2 Department of Nuclear Medicine, The First Affiliated Hospital of Zhengzhou University, Zhengzhou, China; 3 Henan Medical Key Laboratory of Molecular Imaging, Zhengzhou, China

**Keywords:** amplitude of low-frequency fluctuations, cerebral glucose metabolism, functional magnetic resonance imaging, mild cognitive impairment, positron emission tomography, type 2 diabetes mellitus

## Abstract

**Background:**

Type 2 diabetes mellitus (T2DM) is frequently associated with mild cognitive impairment (MCI), significantly impacting patient health, quality of life, and healthcare systems. However, the precise cerebral metabolic and functional alterations resulting from the interaction between T2DM and MCI remain incompletely understood.

**Methods:**

Fifty-four participants were categorized into four groups based on T2DM and MCI status. Hybrid positron emission tomography/functional magnetic resonance imaging (PET/fMRI) was used to assess cerebral glucose metabolism and the amplitude of low-frequency fluctuations (ALFF). A two-way analysis of variance evaluated the main and interactive effects of T2DM and MCI on these measures. Partial correlation analysis examined the associations between clinical variables and the observed metabolic/ALFF abnormalities.

**Results:**

Alterations in cerebral glucose metabolism in T2DM-MCI patients were primarily attributable to T2DM, with significant effects observed in the frontal, occipital, and temporal lobes, as well as subcortical regions. Both main and interactive effects of T2DM and MCI on ALFF were significant. Notably, antagonistic interactions were identified in the right superior frontal gyrus (medial orbital part) and middle frontal gyrus, suggesting that T2DM suppresses spontaneous neural activity in these regions, whereas MCI enhances it. T2DM was the principal factor influencing both metabolic and ALFF changes in the right inferior frontal gyrus (pars orbitalis). Montreal cognitive assessment scores, fasting blood glucose, and 2-hour postprandial blood glucose levels significantly correlated with neural activity alterations in regions affected by the main or interactive effects.

**Conclusion:**

This hybrid PET/fMRI study identifies frontal regions where aberrant metabolic and ALFF patterns are associated with T2DM-related cognitive decline, suggesting their potential as early neuroimaging biomarkers. These findings provide new mechanistic insights and highlight possible therapeutic targets for this comorbidity.

## Introduction

The global rise in diabetes mellitus significantly compromises health and quality of life, while increasing healthcare costs (1). By 2050, over 1.3 billion people worldwide are projected to be affected, predominantly by type 2 diabetes mellitus (T2DM) (1, 2). Chronic hyperglycemia contributes to central nervous system impairment, often beginning as mild cognitive impairment (MCI) and raising the risk of vascular dementia, mixed dementia, and Alzheimer’s disease (AD) (3). Growing evidence also indicates that T2DM independently alters cerebral function (1, 3–5) Understanding the neurobiological mechanisms linking T2DM to cognitive decline is therefore crucial for developing targeted interventions and mitigating neurodegenerative progression (1, 3–5).

Positron emission tomography (PET) and functional magnetic resonance imaging (fMRI) serve as essential, non-invasive modalities for mapping brain function (6, 7). These techniques have increasingly been applied to characterize cerebral alterations in individuals with T2DM (8–10). For instance, Natalia et al. observed reduced glucose metabolism in fronto-temporal regions among middle-aged T2DM patients, a finding correlated with elevated glycated hemoglobin (HbA1c) and homeostatic model assessment of insulin resistance (HOMA-IR) levels (8). Similarly, coexisting MCI and T2DM has been associated with decreased ^18^F-fluorodeoxyglucose (^18^F-FDG) uptake, particularly in the frontal cortex, sensorimotor areas, and striatum (9). Furthermore, Li et al. reported that older adults with T2DM exhibit three notable neural changes: (i) cerebral hypometabolism, (ii) increased integration of functional connectivity (FC), and (iii) enhanced efficiency of information processing, predominantly within networks subserving cognitive function (10).

Multimodal fMRI studies have further delineated spatiotemporal patterns of neural dysregulation in T2DM-related cognitive impairment (1). Degree centrality (DC) analyses indicate disrupted FC density within cognitive control, visual, and memory networks in T2DM patients with MCI (11–13). Compared to healthy controls (HCs), T2DM individuals show increased regional homogeneity (ReHo) in the anterior cingulate cortex (ACC) but decreased ReHo in the medial superior frontal gyrus (14). Seed-based FC analyses further reveal impaired frontal-parietal connectivity, suggesting that deficits in both local and long-range neural synchronization underlie cognitive dysfunction (14). Additionally, alterations in the amplitude of low-frequency fluctuations (ALFF) are consistently reported in T2DM, correlating with impairments in cognition, visual processing, executive function, and emotional regulation (15–17). Furthermore, reduced ALFF and ReHo values have been observed in occipital and postcentral areas, and these reductions were associated with poorer cognitive performance. These functional reductions appear particularly linked to hypoactivity in specific occipital subregions, including the cuneus and lingual gyri (18).

While multimodal PET and fMRI studies have advanced our understanding of cerebral alterations in T2DM and T2DM−MCI, several key gaps remain: (1) The independent and interactive effects of T2DM and MCI on cerebral metabolism and function have not been clearly quantified, partly because few studies include all four essential groups (e.g., inclusion of T2DM-only, MCI-only, T2DM-MCI comorbid, and HCs cohorts); (2) It is still unclear whether the co−occurrence of MCI and T2DM produces synergistic or antagonistic effects on brain metabolism and function; (3) Existing PET−fMRI research is often limited by cohort heterogeneity and inconsistent acquisition protocols, which introduce temporal and state−related confounds and hinder the development of robust spatiotemporal coupling models.

Integrated PET/fMRI represents an advanced neuroimaging approach that allows simultaneous assessment of cerebral metabolism, functional activity, and anatomical structure, effectively overcoming key challenges in multimodal data integration (19, 20) This platform enables a thorough investigation of the independent and interactive effects of T2DM and MCI on brain activity through metabolic-functional coupling, offering valuable insights into pathophysiology and early intervention. ALFF quantifies the power of spontaneous neural activity within low-frequency ranges (e.g., 0.01-0.10 Hz), providing a sensitive and computationally efficient measure of neural dynamics that is responsive to metabolic and pharmacological changes (21, 22). Thus, ALFF serves as a robust methodological framework for examining metabolic-functional coupling in T2DM-related cerebral impairment.

To address these gaps, we employed integrated PET/fMRI to acquire simultaneous multimodal data from four cohorts: HCs, MCI-only, T2DM-only, and T2DM-MCI. Using cerebral glucose metabolism (PET) and ALFF, we assessed the main and interactive effects of T2DM and MCI via a 2×2 factorial two-way analysis of variance (ANOVA). Partial correlation analyses then linked clinical parameters with observed metabolic and ALFF abnormalities. This approach yields a multimodal framework combining metabolic, functional, and clinical data to support early neuroimaging biomarker identification in T2DM-related cognitive decline. Based on prior evidence, we hypothesized that T2DM-MCI interaction alters intrinsic brain activity, which would correlate with clinical and neuropsychological measures. The study aims to clarify how insulin resistance interacts with central pathologies (e.g., amyloid/tau accumulation) in T2DM, elucidating mechanisms of cognitive impairment.

## Materials and methods

### Participants

Ethical approval for the present investigation was obtained from the Ethics Committee of The First Affiliated Hospital of Zhengzhou University (approval reference: 2023-KY-1311-001). Written informed consent was acquired from all participants, who also received compensation for their involvement. Recruitment of T2DM patients occurred within the Department of Endocrinology at the same institution, spanning from December 2023 to July 2024.

Eligibility requirements for the T2DM cohort included: (1) diagnosis conforming to the American Diabetes Association criteria (fasting blood glucose [FBG] ≥ 7.0 mmol/L or 2-hour postprandial blood glucose [2h-PBG] ≥ 11.1 mmol/L during an oral glucose tolerance test [OGTT]) (10, 14, 16); (2) duration of T2DM exceeding one year; (3) age ranging from 18 to 70 years; (4) right-handedness, as ascertained by the Chinese Handedness Inventory (a 10-item assessment), and being a native Chinese speaker; (5) absence of alcohol or substance misuse history; (6) no evidence of cerebral lesions or significant head trauma on T2-weighted FLAIR and T1-weighted MRI sequences; (7) no documented psychiatric or neurological conditions (e.g., depression, dementia); (8) absence of other metabolic disorders (e.g., hyperthyroidism/hypothyroidism); (9) no medications known to affect cerebral function or hemodynamics within three weeks prior to enrollment; (10) absence of MRI contraindications; (11) no history of hypertension or well-controlled blood pressure; (12) no severe diabetes-related complications. HCs met the following criteria: (1) FBG < 6.1 mmol/L and 2h-PG < 7.8 mmol/L (10, 14, 16); and (2) met criteria 3 through 11 as applied to the T2DM participants. All enrolled MCI participants were rigorously assessed to minimize confounding causes of cognitive impairment, with the following exclusions: individuals with neurodegenerative dementia, significant cerebrovascular disease, or current major psychiatric disorders.

Participants were stratified into four groups based on Montreal Cognitive Assessment (MoCA, Beijing version) scores and fasting glucose levels (11, 12, 23) as follows: HCs group: MoCA ≥ 26, FBG < 6.1 mmol/L; MCI group:18 ≤ MoCA < 26, FBG < 6.1 mmol/L; T2DM group: MoCA ≥ 26, FBG ≥ 7.0 mmol/L; T2DM-MCI group: 18 ≤ MoCA < 26, FBG ≥ 7.0 mmol/L.

### Demographics, clinical characteristics, and neurocognitive testing

Baseline demographic and clinical characteristics, including age, sex, body mass index (BMI), and educational attainment, were systematically collected. Fasting blood specimens were obtained between 7:00 and 8:00 AM after an overnight fast of at least 10 hours. Biochemical analyses encompassed serum concentrations of the following: FBG, glycated hemoglobin (HbA1c), fasting insulin (FINS), total cholesterol (TC), triglycerides (TG), high-density lipoprotein (HDL), low-density lipoprotein (LDL), free triiodothyronine (FT3), free thyroxine (FT4), thyroid-stimulating hormone (TSH), serum creatinine (SCR), and uric acid (UA). All assays were performed by the hospital’s clinical laboratory. An OGTT was conducted to measure 2h-PBG, C-peptide (PC-P) and insulin concentrations. Insulin resistance was quantified using the HOMA2 calculator (https://www.dtu.Ox.ac.uk/homacalculator). Additionally, participants completed the Chinese Handedness Questionnaire, Alcohol Use Disorders Identification Test, and Fagerström Test for Nicotine Dependence. MRI safety screening was also performed prior to scanning. The MoCA, which has high sensitivity and specificity for detecting MCI, was used as the primary tool to assess global cognitive function (23). These neuropsychological evaluations, including the MoCA, were conducted by qualified personnel on the same day as the PET/fMRI scan.

### Data acquisition

All subjects adhered to a 12-hour abstinence protocol, refraining from insulin administration, hypoglycemic agents, alcohol, caffeine, tea, and any other substances known to influence cerebral blood flow (CBF). Following a mandatory fasting period of no less than six hours, participants underwent intravenous administration of the radiopharmaceutical agent ^18^F-FDG at a dosage of 3.7 MBq/kg. Post-injection, subjects maintained a supine position with eyes closed for 25–30 minutes within a dimly lit, acoustically isolated environment to minimize sensory stimulation during cerebral metabolic uptake. Prior to imaging commencement, participants voided their bladders and underwent blood glucose verification using a portable glucometer, ensuring levels remained below 11.1 mmol/L. The radiopharmaceutical agent (^18^F-FDG) was synthesized on-site within our institution’s PET/CT Center on the day of examination. Synthesis was performed with a CFN-100 synthesis module integrated with an HM-20 cyclotron (both Sumitomo Heavy Industries, Japan), achieving confirmed radiochemical purity exceeding 95%.

Neuroimaging procedures were conducted at our hospital’s medical imaging center using a 3T Siemens Biograph mMR scanner (Siemens Medical Solutions, Erlangen, Germany) equipped with a standard 16-channel head coil. To enhance participant comfort and reduce motion artifacts, earplugs were provided to reduce scanner noise, and foam padding was used to minimize head movement. PET data acquisition employed list-mode collection over a 50-minute post-injection window, with a scan duration of 10 minutes. Throughout both PET and fMRI scanning sessions, participants maintained closed eyes. PET data reconstruction was performed using 3D ordinary Poisson ordered subsets expectation maximization with the following parameters: three subsets, 21 iterations, 2-mm Gaussian filtering, a 344 × 344 matrix size, 2.0-mm slice thickness, and a voxel resolution of 2.3 mm × 2.3 mm × 2.0 mm.

High-resolution T1-weighted anatomical images were acquired with the following specifications: repetition time (TR) = 2300 ms, echo time (TE) = 2.96 ms, field of view (FOV) = 256 mm × 256 mm, matrix = 256 × 256, 176 slices with 1 mm thickness, and a flip angle of 9°. Prior to subsequent image processing, all datasets were reoriented to a common origin using a center-of-mass approach. Resting-state fMRI (rs-fMRI) data were collected using an echo-planar imaging sequence comprising 270 volumes over a 540-second acquisition period. Sequence parameters included: TR = 2000 ms, TE = 30 ms, FOV = 224 mm × 224 mm, matrix = 64 × 64, 37 slices with 3.5 mm thickness, and a flip angle of 90°.

### PET and MRI data processing

#### PET imaging processing

PET data were processed using SPM12 (implemented in MATLAB 2018a) following these steps (10): (1) The formats of PET and 3D T1-weighted imaging (T1WI) data were converted. (2) PET data were coregistered to each subject’s 3D T1WI structural image. (3) Anatomical structures were segmented. (4) Spatial normalization was performed using the segmentation-derived parameters. (5) Gaussian spatial smoothing was applied with a 10 mm full-width-at-half-maximum (FWHM) kernel (24). (6) The standardized uptake value ratio (SUVr) was calculated for each region, with the whole cerebellum serving as the reference region (25). Visual quality control was performed on all NIFTI and normalized PET images, and those with inadequate quality were discarded prior to analysis.

#### ALFF calculation

Rs-fMRI data pre-processing and ALFF analysis were carried out using the Data Processing and Analysis of Brain Imaging (DPABI, http://rfmri.org/DPABI) toolbox (implemented in MATLAB 2018a). The following preprocessing steps were applied: (1) Data Conversion and Initial Trimming: The formats of rs-fMRI and 3D T1WI data were converted, and the first 10 time points from the rs-fMRI time series were removed. (2) Slice Timing Correction: Differences in acquisition time between slices were corrected. (3) Head Motion Correction: Functional volumes were realigned to correct for subject head motion. (4) Coregistration to Structural Image: Rs-fMRI data were coregistered to the corresponding high-resolution 3D T1WI structural image. (5) Tissue Segmentation: Anatomical structures (gray matter, white matter, cerebrospinal fluid) were segmented from the 3D T1WI image. (6) Nuisance Covariate Regression: To remove non-neuronal fluctuations, signals from cerebrospinal fluid, white matter, the global mean, and the 24 head motion parameters (Friston 24-parameter model) were regressed out. (7) Spatial Normalization: Functional and structural data were normalized into MNI standard stereotactic space using the parameters derived from the segmentation. (8) Spatial Smoothing: Gaussian spatial smoothing was applied with a 6 mm FWHM kernel (21, 22). (9) ALFF Calculation: For each voxel, the time series was transformed to the frequency domain using a Fast Fourier Transform. The square root of the resulting power spectrum was then averaged across the 0.01-0.1 Hz frequency band to obtain the voxel-wise ALFF value (15–17, 21, 22). To facilitate group-level comparisons, each voxel’s ALFF value was divided by the subject’s global mean ALFF. Notably, ALFF calculation was performed without applying band-pass filtering.

### Statistical analysis

#### Demographic and clinical data analysis

Demographic and clinical characteristics were analyzed using SPSS Statistics version 26.0 (http://www.spss.com; Chicago, IL). Normally distributed continuous variables are expressed as mean ± standard deviation, whereas non-normally distributed data are presented as median with the interquartile range. For data which met the assumptions of normality and homogeneity of variance, one-way ANOVA coupled with Bonferroni *post-hoc* tests was employed. For data which violated these assumptions, the Kruskal-Wallis H test was applied. As the expected frequencies in some contingency table cells were below 5, group differences in sex distribution were evaluated using the Fisher-Freeman-Halton exact test for categorical variables (26). To mitigate potential confounding effects, age, sex, and years of education were included as covariates in all subsequent statistical analyses.

#### PET and fMRI data analysis

Given the differences in spatial resolution and signal properties between PET and rs-fMRI data, this study employed differentiated analytical strategies to optimize the efficacy of each modality. First, whole-brain voxel-wise analysis was performed on the PET images, which have a relatively high signal-to-noise ratio, to unbiasedly explore brain regions with altered glucose metabolism associated with T2DM (19). Subsequently, the significant clusters identified in the PET analysis were defined as regions of interest (ROIs), and the mean ALFF values within these ROIs were extracted for group-level statistical comparison (27). This analysis pipeline, driven by PET findings to guide ALFF verification, aims to ensure anatomical consistency across modalities and enhance the statistical power of the ALFF analysis under specific hypotheses.

To investigate the interaction effect of MCI × T2DM on cerebral glucose metabolism, along with their main effects, a voxel-wise two-way ANOVA was conducted in SPM12, with age, sex, and years of education included as covariates. Statistical significance was determined using a false discovery rate (FDR) correction with a threshold of *p* < 0.05. The SUVr values from significant brain regions, which were identified using the Automated Anatomical Labeling 3 (AAL3) atlas template (28), were extracted for subsequent analysis. To examine functional activity in these metabolically altered regions, ALFF values were then extracted from the same clusters. A follow-up two-way ANOVA was performed in SPSS 26.0 to assess the MCI × T2DM interaction and main effects on ALFF, covarying for age, sex, and years of education. The significance of these effects was assessed with an FDR correction for multiple comparisons, set at *p* < 0.05.

### Brain-behavior partial correlation analysis

Brain regions showing significant interaction or main effects in the two-way ANOVA were identified. The average SUVr and ALFF values within these regions were extracted for each participant. We then performed partial correlation analyses to assess the associations of both SUVr and ALFF values with clinical measures (e.g., MoCA scores) and blood biomarkers (e.g., HbA1c), adjusting for age, sex, and years of education. Statistical significance for these correlations was set at *p* < 0.05, corrected for multiple comparisons across all tests using the false discovery rate (FDR) method.

## Results

### Demographic and clinical data

Sixty-nine participants were enrolled and underwent ^18^F-FDG PET/fMRI scans. After excluding 15 individuals who did not meet the inclusion criteria or exhibited any abnormal imaging findings, 54 subjects were included in the final analysis. The study population comprised four groups: HCs (n=14), MCI (n=13), T2DM (n=12), and T2DM-MCI (n=15). **Table 1** presents the demographic and clinical characteristics across these groups. There were significant differences among groups in age, years of education, FBG, HbA1c, 2h-PG, MoCA, and Mini-Mental State Examination scores. No statistically significant differences were found for other demographic or clinical parameters.

**Table 1:** Participant demographics, clinical characteristics, and neuropsychological test scores.

Variables	HCs (n =14)	MCI (n = 13)	T2DM (n = 12)	T2DM-MCI (n = 15)	F value	*p* value
Age (years)	45.14 ± 10.22	56.77 ± 11.90	52.17 ± 9.21	55.80 ± 5.78	4.33^a^	0.009
Sex (male/female)	8/6	7/6	8/4	13/2	4.37^c^	0.226
Education (years)	19.00 (3.00)	9.00 (3.00)	12.00 (6.25)	9.00 (3.00)	26.67^b^	<0.001
BMI (kg/m^2^)	24.18 ± 2.94	23.88 ± 2.41	25.00 ± 2.08	26.39 ± 3.39	2.31^a^	0.088
FBG (mmol/L)	5.26 ± 0.32	5.42 ± 0.44	6.95 ± 1.34	6.76 (1.10)	30.95^b^	<0.001
HbA1c (%)	5.43 ± 0.31	5.70 ± 0.31	7.30 ± 0.95	7.40 (1.30)	40.02^b^	<0.001
FCP(ng/mL)	2.19 ± 0.39	2.55 ± 11.90	2.25 ± 0.92	2.40 ± 0.69	0.71^a^	0.549
FINS (μU/mL)	7.72 ± 2.31	9.31 ± 4.15	9.72 ± 6.82	8.66 ± 4.43	0.47^a^	0.703
HOMA2-IR	1.80 ± 0.52	2.25 ± 1.06	3.16 ± 2.41	2.26 ± 1.20	5.45^b^	0.142
TC (mmol/L)	4.59 ± 1.01	8.99 ± 13.61	4.81 (1.33)	4.68 ± 1.03	0.409^b^	0.938
TG (mmol/L)	1.07 (0.90)	2.13 ± 1.30	1.48 ± 0.61	1.90 ± 0.82	3.699^b^	0.296
HDL (mmol/L)	1.31 ± 0.27	1.34 ± 0.34	1.15 ± 0.28	1.21 ± 0.22	1.36^a^	0.267
LDL (mmol/L)	2.65 ± 0.84	2.75 ± 0.63	3.09 ± 1.08	2.71 ± 0.92	0.386^a^	0.764
FT3 (pmol/L)	4.83 ± 0.51	5.36 ± 0.66	4.99 ± 0.81	4.91 ± 0.59	0.976^a^	0.412
FT4 (pmol/L)	9.90 (2.27)	10.98 ± 2.32	11.73 ± 1.81	12.55 ± 1.94	7.402^b^	0.06
TSH (μIU/mL)	2.21 (1.17)	1.83 ± 0.65	2.10 ± 1.36	2.75 ± 1.70	2.259^b^	0.52
2h-PBG (mmol/L)	6.72 ± 1.81	6.72 (1.22)	14.47 ± 4.14	16.15 ± 4.25	38.38^b^	<0.001
2h-FINS (pmol/L)	54.35 ± 29.25	54.70 (34.55)	65.23 ± 40.76	55.44 ± 39.22	0.78^b^	0.855
2h-FCP(ng/ml)	8.94 ± 2.53	10.39 ± 2.71	9.29 ± 3.89	8.22 ± 3.74	1.05^a^	0.378
MoCA (scores)	27.57 ± 1.45	22.54 ± 1.66	26.50 (2.00)	23.00 (1.00)	40.86^b^	<0.001
MMSE (scores)	29.00 (1.00)	26.00 ± 2.08	28.00 (2.75)	25.27 ± 1.75	22.74^b^	<0.001

Normally distributed data are expressed as mean ± standard deviation (SD). Non-normally distributed data are presented as median and interquartile range (IQR). Between-group differences were assessed using one-way ANOVA for normally distributed data (a), the Kruskal-Wallis H test for non-normally distributed data (b), and the Fisher-Freeman-Halton exact test for sex distribution (c). HCs, healthy controls; T2DM, type 2 diabetes mellitus; MCI, mild cognitive impairment; BMI, body mass index; FBG, fasting blood glucose; 2h-PBG, 2-hour postprandial blood glucose; HbA1c, hemoglobin A1c; FCP, fasting C-peptide; FINS, fasting insulin; HOMA2-IR, homeostasis model assessment insulin resistance; TC, total cholesterol; TG, triglyceride; HDL, high-density lipoprotein; LDL, low-density lipoprotein; FT3, free triiodothyronine; FT4, free thyroxine; TSH, thyroid-stimulating hormone; MoCA, montreal cognitive assessment (Beijing version); MMSE, mini-mental state examination.

### PET analysis

**Figure 1:**
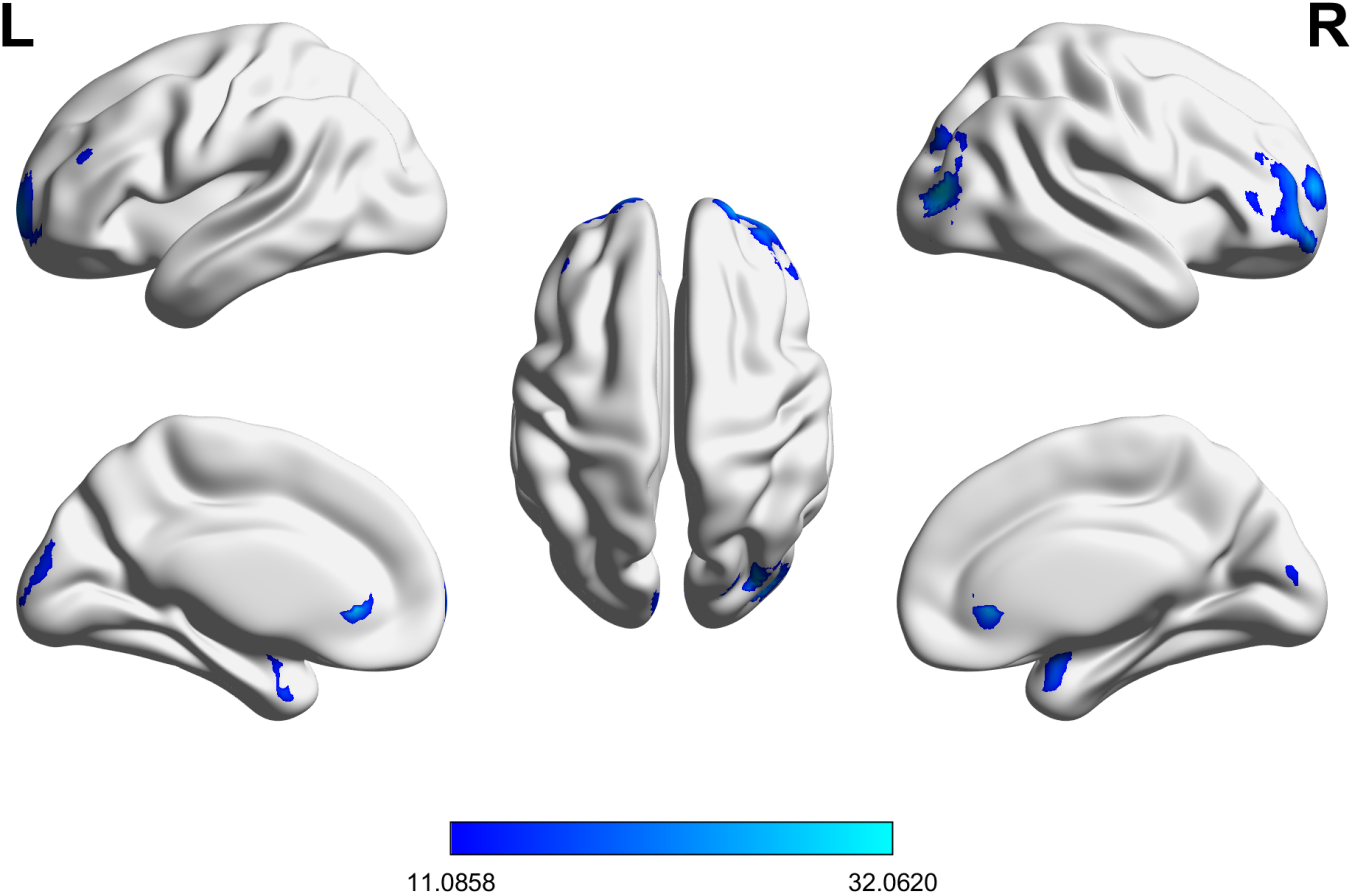
Brain regions showing a significant main effect of type 2 diabetes on cerebral glucose metabolism. The statistical threshold was set at a cluster-level *p* < 0.05, corrected for multiple comparisons using the false discovery rate (FDR). The color bar represents the F-value scale. L, left; R, right.

The two-way ANOVA revealed no significant MCI × T2DM interaction nor a main effect of MCI. However, a significant main effect of T2DM was observed in multiple brain regions (*p <* 0.05, FDR corrected). The mean SUVr differences were defined as the values in the T2DM and T2DM-MCI groups relative to those in the HC and MCI groups. Decreases in SUVr were observed across four main clusters, encompassing the following regions: right middle frontal gyrus (MFG), right superior frontal gyrus-dorsolateral (SFG), right inferior frontal gyrus (triangular, opercular, and orbital parts; IFGtriang, IFGoperc, IFGorb), right middle/superior/inferior occipital gyri (MOG, SOG, IOG), right cuneus, right angular gyrus (ANG), right calcarine fissure and surrounding cortex (CAL), left SFG, right MOG, left superior frontal gyrus-medial (SFGmedial), left IFGtriang, left superior frontal gyrus-medial orbital (PFCventmed), left cuneus, left CAL, left SOG, left MOG, and right cuneus. In contrast, increased SUVr was identified within three clusters, including left parahippocampal gyrus (PHG), left amygdala, left temporal pole (superior and middle temporal gyri; TPOsup, TPOmid), left inferior temporal gyrus (ITG), right amygdala, right TPOsup, right PHG, bilateral subgenual and pregenual ACC (ACCsub, ACCpre), right PFCventmed, and bilateral olfactory cortex (OLF). These metabolic alterations are associated with T2DM, as indicated by the significant main effect. The spatial distributions of the altered cerebral glucose metabolism are illustrated in **Figure 1**, with detailed information on the peak metabolic differences provided in **Table 2**.

**Table 2:** Main effect of T2DM on cerebral glucose metabolism from two-way ANOVA.

Brain regions	Location in brain	Whole cluster size	Cluster size	MNI coordinates			Peak F-value	Mean SUVr difference
				x	y	z		
R Middle frontal gyrus	Frontal Lobe	1504	668	26	60	-12	26.19	<0
R Superior frontal gyrus-dorsolateral	Frontal Lobe		535	28	60	-8	28.33	<0
R Inferior frontal gyrus-triangular part	Frontal Lobe		234	46	38	12	20.08	<0
R Inferior frontal gyrus-opercular part	Frontal Lobe		25	48	22	32	16.07	<0
R Inferior frontal gyrus pars orbitalis	Frontal Lobe		15	44	48	-2	15.52	<0
R Middle occipital gyrus	Occipital Lobe	782	467	34	-84	12	32.06	<0
R Superior occipital gyrus	Occipital Lobe		128	30	-84	26	23.27	<0
R Inferior occipital gyrus	Occipital Lobe		108	38	-84	-2	28.51	<0
R Cuneus	Occipital Lobe		56	4	-84	16	13.12	<0
R Angular gyrus	Parietal Lobe		11	46	-72	32	11.76	<0
R Calcarine fissure and surrounding cortex	Occipital Lobe		10	26	-94	2	14.34	<0
L Superior frontal gyrus-dorsolateral	Frontal Lobe	519	311	-18	64	0	23.74	<0
R Middle occipital gyrus	Occipital Lobe		122	-30	58	-8	16.75	<0
L Superior frontal gyrus-medial	Frontal Lobe		35	-16	66	0	22.86	<0
L Inferior frontal gyrus-triangular part	Frontal Lobe		33	-46	38	12	11.66	<0
L Superior frontal gyrus-medial orbital	Frontal Lobe		18	-14	66	-2	20.08	<0
L Cuneus	Occipital Lobe	357	182	0	-86	16	16.86	<0
L Calcarine fissure and surrounding cortex	Occipital Lobe		102	2	-88	14	15.59	<0
L Superior occipital gyrus	Occipital Lobe		31	-10	-92	18	12.77	<0
L Middle occipital gyrus	Occipital Lobe		21	-12	-100	2	12.71	<0
R Cuneus	Occipital Lobe		14	4	-84	16	13.12	<0
L Parahippocampal gyrus	Subcortical Structures	189	51	-28	2	-32	16.65	>0
L Amygdala	Subcortical Structures		48	-30	0	-28	15.52	>0
L Temporal pole: superior temporal gyrus	Temporal Lobe		37	-36	4	-22	13.01	>0
L Temporal pole: middle temporal gyrus	Temporal Lobe		35	-16	10	-38	23.04	>0
L Inferior temporal gyrus	Temporal Lobe		11	-32	4	-36	14.09	>0
R Amygdala	Subcortical Structures	206	114	30	4	-26	21.20	>0
R Temporal pole: superior temporal gyrus	Temporal Lobe		56	32	4	-24	19.99	>0
R Parahippocampal gyrus	Subcortical Structures		36	28	4	-26	18.98	>0
R Anterior cingulate cortex-subgenual	Frontal Lobe	306	104	2	28	0	27.60	>0
L Anterior cingulate cortex-subgenual	Frontal Lobe		65	-4	30	0	31.35	>0
R Superior frontal gyrus-medial orbital	Frontal Lobe		45	12	36	-10	18.78	>0
R Anterior cingulate cortex-pregenual	Frontal Lobe		17	18	42	2	14.16	>0
L Olfactory cortex	Temporal Lobe		14	-6	30	-2	26.99	>0
R Olfactory cortex	Temporal Lobe		10	6	28	-2	23.01	>0

The statistical threshold was set at *p* < 0.05 with false discovery rate correction. The mean SUVr difference represents the contrast between the combined T2DM groups (T2DM and T2DM-MCI) and the combined non-T2DM groups (healthy controls and MCI). L, Left; R, Right; T2DM, type 2 diabetes mellitus; MCI, mild cognitive impairment; SUVr, the standardized uptake value ratio; ANOVA, analysis of variance.

### ALFF analysis

**Figure 2:**
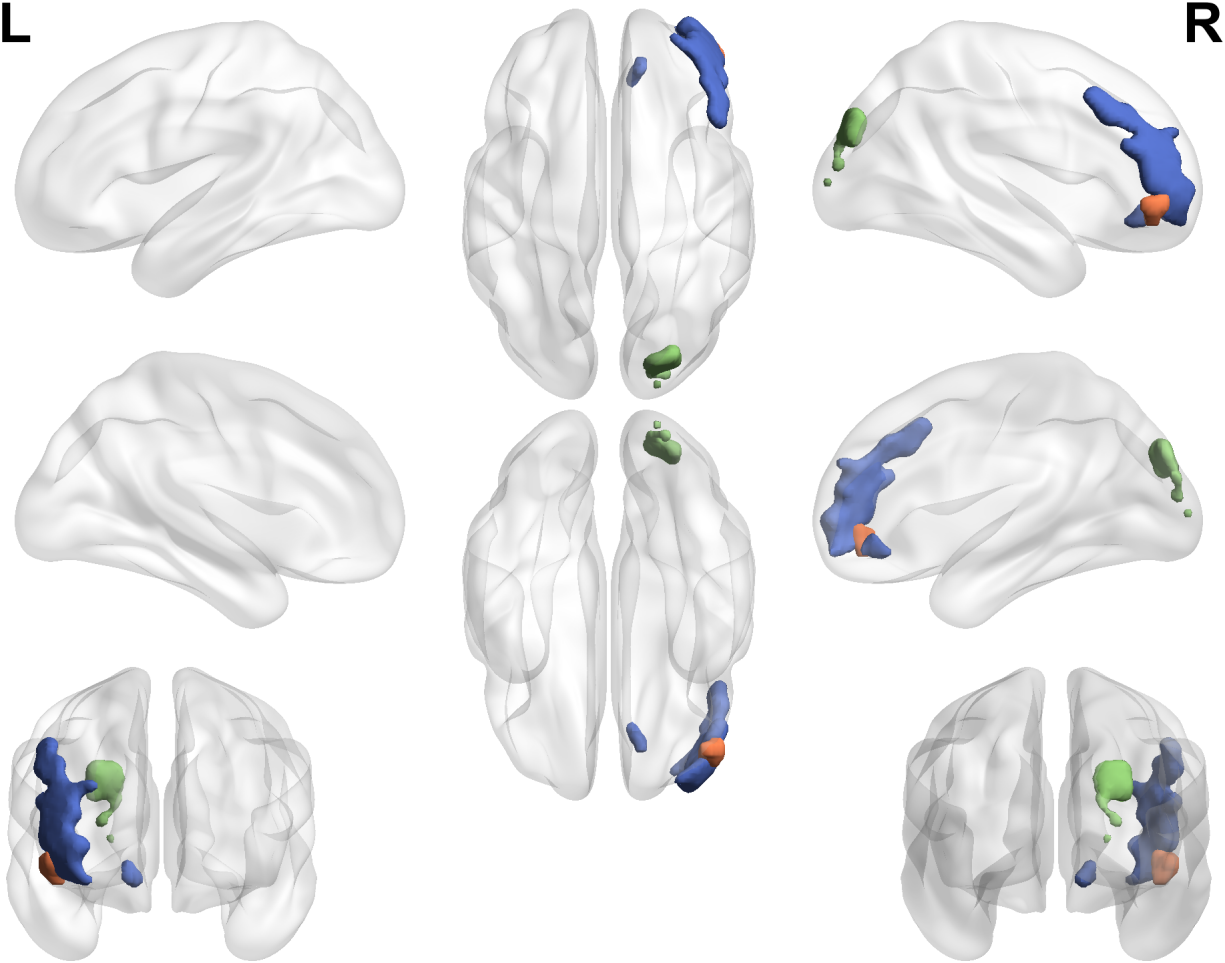
Brain regions showing significant main effects of and interaction between MCI and T2DM on the amplitude of low-frequency fluctuations. Main effects of MCI, main effects of T2DM, and MCI × T2DM interaction effects are shown in green, orange, and blue, respectively. The statistical threshold was set at *p* < 0.05, corrected for multiple comparisons using the false discovery rate (FDR). L, left; R, right; T2DM, type 2 diabetes mellitus; MCI, mild cognitive impairment.

**Figure 3:**
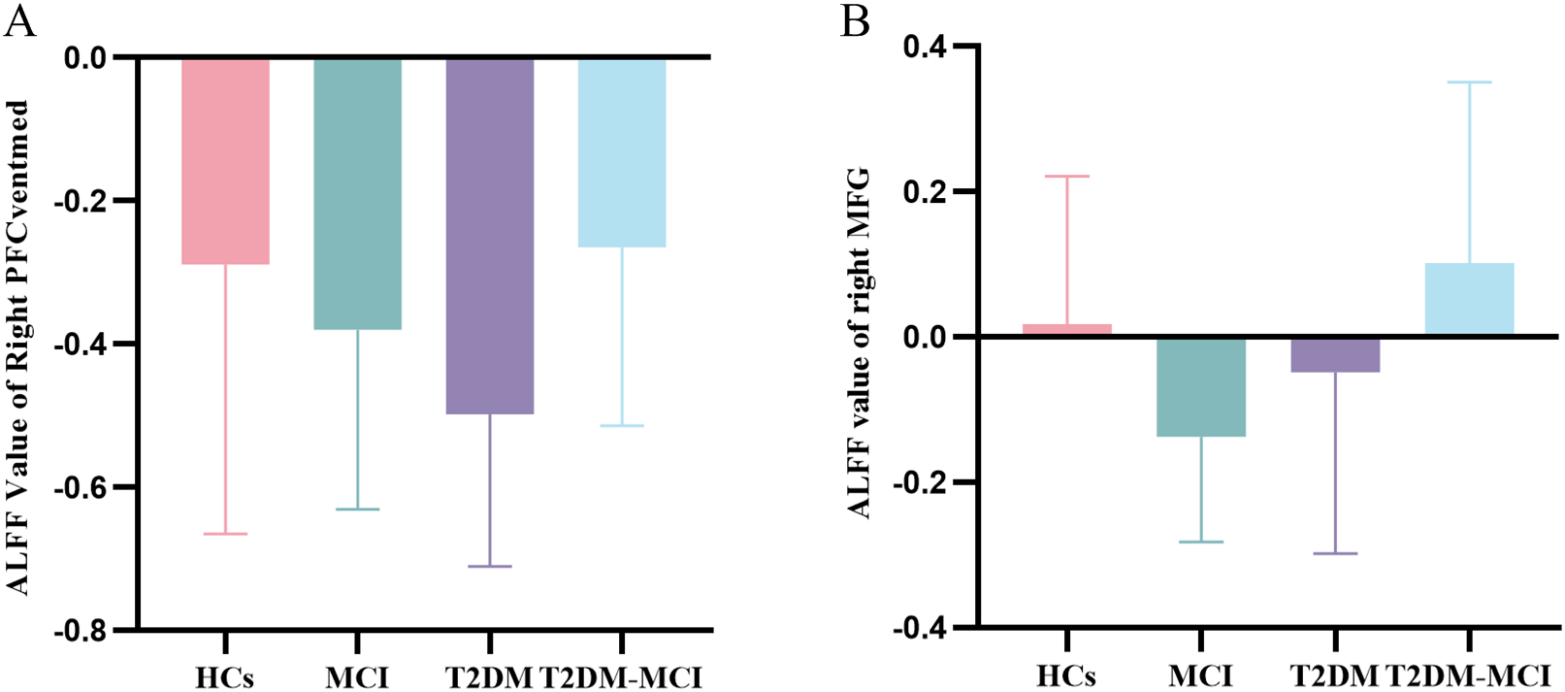
ALFF values in the right PFCventmed **(A)** and right MFG **(B)**, showing the interaction between MCI and T2DM across the four participant groups. ALFF, amplitude of low-frequency fluctuations; HCs, healthy controls; T2DM, type 2 diabetes mellitus; MCI, mild cognitive impairment; PFCventmed, medial orbital part of the superior frontal gyrus; MFG, middle frontal gyrus.

A two-way ANOVA on ALFF values, extracted from regions with altered cerebral glucose metabolism, revealed significant main effects of both MCI and T2DM, as well as a significant MCI × T2DM interaction (*p* < 0.05, FDR-corrected). The distribution of these brain regions is shown in **Figure 2**. The main effect of MCI was characterized by decreased ALFF in the right SOG, indicating lower activity in the groups with MCI (MCI and T2DM-MCI) compared to those without (HC and T2DM). The main effect of T2DM was characterized by increased ALFF in the right IFGorb, indicating higher activity in the groups with T2DM (T2DM and T2DM-MCI) compared to those without (HC and MCI). The MCI × T2DM interaction was observed in the right PFCventmed and the right MFG. **Figure 3** illustrates the ALFF values in these regions across the four groups. It shows that individuals with MCI had lower ALFF compared to HCs, whereas individuals with both T2DM and MCI demonstrated higher ALFF than those with T2DM alone. These opposing patterns indicate an antagonistic interaction effect of MCI and T2DM on neural activity in the right PFCventmed and MFG. **Table 3** provides detailed coordinates for these significant clusters.

**Table 3:** Main and interaction effects of T2DM and MCI on ALFF from two-way ANOVA.

Brain regions	Main effect of MCI				Main effect of T2DM				Interaction effect of MCI×T2DM		
	F(1, 47)	P	Partial η^2^	Mean ALFF difference	F(1, 47)	P	Partial η^2^	Mean ALFF difference	F(1, 47)	P	Partial η2
R Middle frontal gyrus	0.144	0.706	0.003		0.591	0.446	0.012		5.251	0.026*	0.1
R Superior frontal gyrus-dorsolateral	0.008	0.928	0		1.382	0.246	0.029		1.454	0.234	0.03
R Inferior frontal gyrus-triangular part	0.004	0.952	0		0.639	0.428	0.013		0.003	0.953	0
R Inferior frontal gyrus-opercular part	0.058	0.811	0.001		0.345	0.56	0.007		0.715	0.402	0.015
R Inferior frontal gyrus pars orbitalis	0.86	0.359	0.018		4.342	0.043*	0.085	>0	1.032	0.315	0.021
R Middle occipital gyrus	0.57	0.454	0.012		0.016	0.901	0		0.386	0.537	0.008
R Superior occipital gyrus	8.601	0.005*	0.155	<0	0.014	0.907	0		0.186	0.668	0.004
R Inferior occipital gyrus	0.119	0.732	0.003		0.001	0.975	0		0.708	0.404	0.015
R Cuneus	2.034	0.16	0.041		0.193	0.663	0.004		0.011	0.916	0
R Angular gyrus	1.961	0.168	0.04		1.029	0.316	0.021		0.059	0.81	0.001
R Calcarine fissure and surrounding cortex	0.574	0.453	0.012		0.117	0.734	0.002		0.027	0.87	0.001
L Superior frontal gyrus-dorsolateral	0.008	0.928	0		1.382	0.246	0.029		1.454	0.234	0.03
R Middle occipital gyrus	0.1	0.754	0.002		1.397	0.243	0.029		3.606	0.064	0.071
L Superior frontal gyrus-medial	0.021	0.886	0		0.289	0.593	0.006		0.552	0.461	0.012
L Inferior frontal gyrus-triangular part	0.001	0.975	0		0.252	0.618	0.005		2.374	0.13	0.048
L Superior frontal gyrus-medial orbital	0.888	0.351	0.019		0.062	0.805	0.001		1.18	0.283	0.024
L Cuneus	0.029	0.866	0.001		1.193	0.28	0.025		0.036	0.849	0.001
L Calcarine fissure and surrounding cortex	0.075	0.786	0.002		0.702	0.406	0.015		0.164	0.687	0.003
L Superior occipital gyrus	0.018	0.894	0		2.122	0.152	0.043		0.652	0.424	0.014
L Middle occipital gyrus	0	0.997	0		0.748	0.391	0.016		0.642	0.427	0.013
R Cuneus	0.058	0.81	0.001		0	0.992	0		0.362	0.55	0.008
L Parahippocampal gyrus	0.031	0.861	0.001		1	0.323	0.021		2.96	0.092	0.059
L Amygdala	0.732	0.396	0.015		1.372	0.247	0.028		0.742	0.393	0.016
L Temporal pole: superior temporal gyrus	0.308	0.581	0.007		0.011	0.915	0		1.151	0.289	0.024
L Temporal pole: middle temporal gyrus	0.039	0.844	0.001		1.693	0.199	0.035		2.703	0.107	0.054
L Inferior temporal gyrus	0.91	0.345	0.019		0.305	0.583	0.006		0.931	0.34	0.019
R Amygdala	0.022	0.883	0		1.659	0.204	0.034		0.39	0.535	0.008
R Temporal pole: superior temporal gyrus	0.033	0.856	0.001		0.293	0.591	0.006		0.025	0.875	0.001
R Parahippocampal gyrus	0.078	0.781	0.002		0.168	0.684	0.004		0.004	0.949	0
R Anterior cingulate cortex-subgenual	0.254	0.617	0.005		0.097	0.757	0.002		3.065	0.087	0.061
L Anterior cingulate cortex-subgenual	0.39	0.535	0.008		0.185	0.669	0.004		1.914	0.173	0.039
R Superior frontal gyrus-medial orbital	0.773	0.384	0.016		0.044	0.835	0.001		4.6	0.037*	0.089
R Anterior cingulate cortex-pregenual	0.086	0.77	0.002		2.041	0.16	0.042		0.012	0.913	0
L Olfactory cortex	0.342	0.562	0.007		0.016	0.901	0		1.149	0.289	0.024
R Olfactory cortex	0.726	0.398	0.015		0.004	0.948	0		0.563	0.457	0.012

The statistical threshold was set at *p* < 0.05, corrected for multiple comparisons using the false discovery rate (FDR). The main effect of MCI was defined by contrasting the combined MCI and T2DM-MCI groups against the combined HC and T2DM groups. Similarly, the main effect of T2DM was defined by contrasting the combined T2DM and T2DM-MCI groups against the combined HCs and MCI groups. L, Left; R, Right; HCs, healthy controls; T2DM, type 2 diabetes mellitus; MCI, mild cognitive impairment; ALFF, amplitude of low-frequency fluctuations; ANOVA, analysis of variance. *p < 0.05.

### Brain-behavior partial correlation analysis

**Figure 4:**
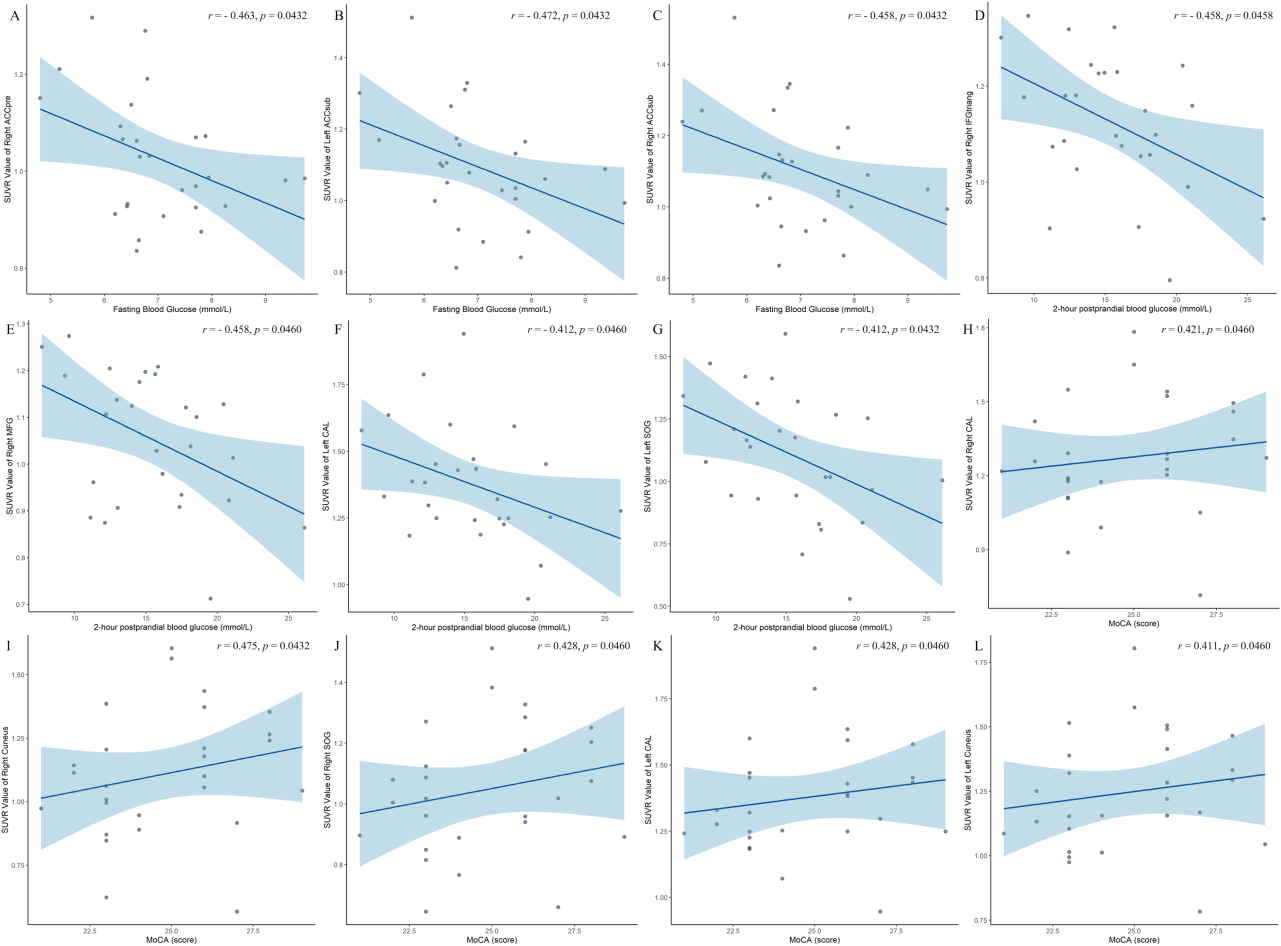
**(A–L)** Correlations between clinical variables and cerebral glucose metabolism (SUVr) in T2DM and T2DM-MCI groups. Scatter plots depict the relationship between clinical measures (FBG, 2h-PBG, MoCA) and SUVr in brain regions that showed a significant main effect of T2DM. T2DM, type 2 diabetes mellitus; MCI, mild cognitive impairment; SUVr, standardized uptake value ratio; FBG, fasting blood glucose; 2h-PBG, 2-hour postprandial blood glucose; MoCA, Montreal Cognitive Assessment; ACCpre, anterior cingulate cortex-pregenual; ACCsub, anterior cingulate cortex-subgenual; IFGtriang, inferior frontal gyrus-triangular part; MFG, middle frontal gyrus; CAL, calcarine fissure and surrounding cortex; SOG, superior occipital gyrus.

**Figure 5:**
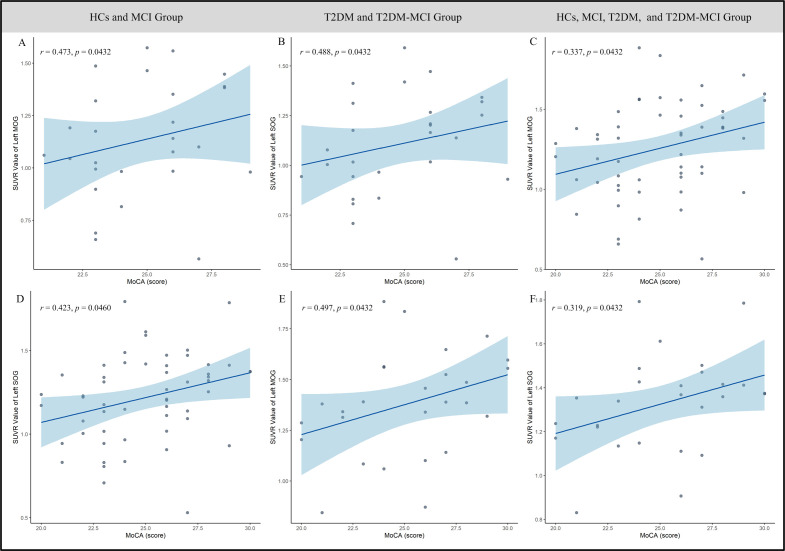
**(A–F)** Scatter plots of the association between MoCA scores and SUVr in the left SOG and MOG. This analysis demonstrates that T2DM significantly influences the relationship between cognitive performance and cerebral glucose metabolism. MoCA, Montreal Cognitive Assessment; SUVr, standardized uptake value ratio; SOG, superior occipital gyrus; MOG, middle occipital gyrus; HC, healthy controls; T2DM, type 2 diabetes mellitus; MCI, mild cognitive impairment.

Based on brain regions identified through two-way ANOVA of PET data, significant negative correlations emerged between FBG and SUVr values in T2DM and T2DM-MCI subjects for the right ACCpre (*r* = -0.463, *p* = 0.0432, **Figure 4A**), left ACCsub (*r* = -0.472, *p* = 0.0432, **Figure 4B**), and right ACCsub (*r* = -0.458, *p* = 0.0432, **Figure 4C**). Additionally, in the same groups, 2h-PBG showed significant negative correlations with SUVr values in the right IFGtriang (*r* = -0.458, *p* = 0.0458, **Figure 4D**), right MFG (*r* = -0.458, *p* = 0.0460, **Figure 4E**), left CAL (*r* = -0.412, *p* = 0.0460, **Figure 4F**), and left SOG (*r* = -0.412, *p* = 0.0432, **Figure 4G**). Conversely, MoCA scores showed significant positive correlations with SUVr values in the right CAL (*r* = 0.421, *p* = 0.0460, **Figure 4H**), right cuneus (*r* = 0.475, *p* = 0.0432, **Figure 4I**), right SOG (*r* = 0.428, *p* = 0.0460, **Figure 4J**), left CAL (*r* = 0.428, *p* = 0.0460, **Figure 4K**), and left cuneus (*r* = 0.411, *p* = 0.0460, **Figure 4L**). However, these correlations were not statistically significant in the HCs and MCI groups. Of particular note, in T2DM and T2DM-MCI subjects, MoCA scores also exhibited significant positive correlations with SUVr values in the left MOG (*r* = 0.488, *p* = 0.0432, **Figure 5B**) and left SOG (*r* = 0.497, *p* = 0.0432, **Figure 5E**), as well as in the HCs and HC-MCI groups (MOG: *r* = 0.337, *p* = 0.0432, **Figure 5A**; SOG: *r* = 0.319, *p* = 0.0432, **Figure 5D**), or across all subjects (MOG: *r* = 0.473, *p* = 0.0432, **Figure 5C**; SOG: *r* = 0.423, *p* = 0.0460, **Figure 5F**).

**Figure 6:**
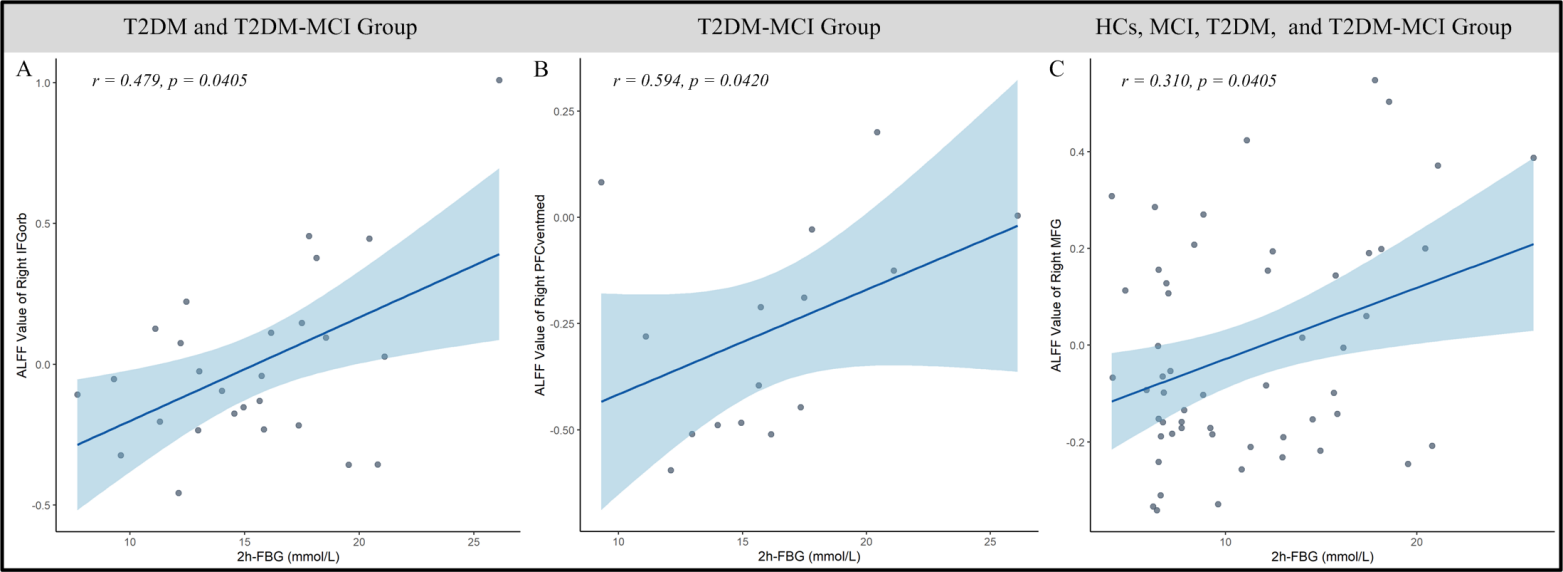
Relationships between 2h-PBG and ALFF. **(A)** Correlation in the right IFGorb, a region showing a significant main effect of T2DM. **(B, C)** Correlations in the right PFCventmed **(B)** and right MFG **(C)**, regions showing a significant MCI × T2DM interaction effect. 2h-PBG, 2-hour fasting blood glucose; ALFF, amplitude of low-frequency fluctuations; T2DM, type 2 diabetes mellitus; MCI, mild cognitive impairment; IFGorb, inferior frontal gyrus, orbital part; PFCventmed, medial orbital prefrontal cortex; MFG, middle frontal gyrus.

Several positive correlations were observed between regional ALFF and 2h-PBG levels in specific subgroups. In T2DM and T2DM-MCI subjects, values in the right IFGorb were significantly correlated with 2h-PBG (*r* = 0.479, *p* = 0.0405, **Figure 6A**). Within the T2DM-MCI subgroup, ALFF in the right PFCventmed was positively correlated with 2h-PBG (*r* = 0.594, *p* = 0.042, **Figure 6B**). In addition, across all participants, ALFF in the right MFG also demonstrated a positive correlation with 2h-PBG levels (*r* = 0.31, *p* = 0.0405, **Figure 6C**).

## Discussion

Using hybrid PET/fMRI, we examined the independent and interactive effects of T2DM and MCI, and the relationships between clinical measures and altered metabolic/ALFF patterns. Key findings include: (1) Cerebral glucose metabolism changes in T2DM-MCI individuals were mainly attributable to T2DM, involving frontal, occipital, temporal, and subcortical regions. (2) Both T2DM and MCI exhibited significant main effects on ALFF, with a crossover interaction in the right medial orbital prefrontal cortex and middle frontal gyrus, where their effects on neural activity were opposing. (3) In the right orbital inferior frontal gyrus, T2DM was the principal factor affecting both metabolism and ALFF. (4) Clinical variables (MoCA scores, FBG, and 2h-PBG) correlated significantly with the observed metabolic and ALFF alterations in regions showing the main or interactive effects.

### T2DM-driven alterations in cerebral glucose metabolism

This investigation demonstrates that diminished cerebral glucose metabolism associated with T2DM occurs primarily in the frontal lobe (comprising bilateral SFG, bilateral IFGtriang, left SFGmedial, right MFG, right IFGoperc, and right IFGorb), the occipital lobe (encompassing bilateral SOG, bilateral MOG, bilateral cuneus, bilateral CAL, and right IOG), and the right ANG. Conversely, we found elevated metabolic activity in clusters located in the dorsomedial frontal lobe (including bilateral PFCventmed, bilateral ACCsub, and right ACCpre), subcortical structures (bilateral PHG and bilateral amygdala), and the temporal lobe (bilateral OLF, left TPOsup, left TPOmid, left ITG, and right TPOsup).

Consistent with prior reports of reduced occipital perfusion in T2DM and its link to visuospatial and memory deficits (29, 30), our study found positive correlations between MoCA scores and glucose metabolism (SUVr) in occipital regions. Specifically, positive associations were observed in the left MOG and SOG across all participants, and in the bilateral cuneus, CAL, and right SOG within T2DM groups. The ANG, a key hub for integrative cognition and a potential biomarker for T2DM-related cognitive decline (31), shows reduced glucose metabolism linked to higher Alzheimer’s disease risk in pre-diabetic and T2DM populations (32). Furthermore, impaired frontal metabolism, particularly in the right IFGtriang and right MFG, was negatively correlated with 2h-PBG, reinforcing the biological basis for executive dysfunction in T2DM (8, 9). Together, these regionally specific metabolic decreases help explain the neurobiological underpinnings of domain-specific cognitive impairment in T2DM.

Amygdala metabolic activity demonstrates significant correlations with FBG and HbA1c levels, with elevated activity predicting increased T2DM risk (33, 34). Post-interventional metabolism reductions in the amygdala have been documented in select T2DM cohorts (33). Among amyloid PET-positive participants, the diabetic group exhibited significantly higher tau PET SUVr in the medial temporal lobe, including the bilateral amygdala, hippocampus, and entorhinal cortex (frontal part of the PHG), compared to the non-diabetic group (25). Similarly, Queralt et al. found that patients with T2DM exhibited increased metabolic activity in the left amygdala and hippocampus at baseline compared to the same individuals during the hyperinsulinemic-euglycemic clamp procedure (35). Compared with HCs, patients with T2DM demonstrated increased CBF (hyperperfusion) in the ACC, which is a key region for higher-order cognitive control. The WMS-III Text Recall subtest scores correlated significantly with glucose metabolism in several regions, including the bilateral subgenual ACC, orbital prefrontal cortex, right PHG, and the right ACC proper (36). Moreover, FBG showed significant positive correlations with SUVr values in bilateral ACCsub and right ACCpre across T2DM and T2DM-MCI groups. Previous studies in T2DM patients report reduced brain volume, gray matter density, and ALFF in the temporal lobe, and these brain anomalies have also been linked to reduced cognitive performance (37, 38). In contrast, increased CBF values in the left MTG have been observed specifically in patients with diabetic retinopathy (37, 38). Collectively, increased glucose metabolism in these regions may represent a compensatory neural response to offset cognitive and emotional impairment in T2DM.

### Independent and interactive effects of MCI and T2DM on ALFF

The primary pathophysiological effect of MCI is characterized by reduced ALFF, predominantly localized to the right SOG. As a critical region within the occipital lobe, the SOG plays a central role in visuospatial processing, attentional regulation, and the integration of multisensory and higher-order cognitive inputs (39–41). This finding aligns with reports of decreased ALFF (41, 42) and reduced cerebral blood flow (43) in occipital regions in MCI (42, 43). Additionally, individuals with leukoaraiosis and cognitive impairment demonstrate significantly lower ALFF in the right SOG compared to those with leukoaraiosis alone (39). Meta-analytic evidence further indicates reduced regional cerebral blood flow in the SOG in MCI populations (44). These findings suggest that MCI disrupts both local energy metabolism and spontaneous neural activity in the right SOG, potentially contributing to the visual processing deficits commonly observed in MCI and AD (45). Adding to this evidence, Zhang et al. reported that enhanced functional connectivity between the thalamus and SOG is associated with early cognitive impairment in individuals with insomnia (41). Our results thus highlight a neuropathological mechanism that may underlie visual and cognitive dysfunction in MCI.

In contrast, T2DM is primarily associated with elevated ALFF, most notably in the right orbital part of the inferior frontal gyrus (IFGorb). This region, situated within the prefrontal cortex, is key for cognitive control processes such as impulse inhibition and executive function (46). Our observations align with those of Li et al. and Luo et al., who also documented significantly increased ALFF in the right IFGorb among T2DM patients compared to HCs (47, 48). Structural abnormalities, including reduced gray matter volume in the right IFG and decreased cortical thickness in bilateral IFGorb, have been reported in T2DM cohorts (1, 49) and are closely linked to aberrant neural activity (1). Collectively, these results indicate concurrent impairments in the structure and function of the right IFGorb in T2DM (1). Previous research suggests that the most anterior portion of the right IFGorb is specifically engaged in higher-order inhibitory control, distinguishing it from adjacent subregions (50). Elevated ALFF in this area may therefore be a biomarker for cognitive control deficits in T2DM.

The interaction between MCI and T2DM exhibits an antagonistic effect on ALFF within the right PFCventmed and the MFG. These regions, integral nodes of the default mode network (DMN), are implicated in reward processing, emotion regulation, and decision-making (1, 51, 52). Patients with MCI have been reported to exhibit increased ALFF in anterior DMN regions, such as the bilateral PFCventmed and left MFG (48, 53). In contrast, a recent meta-analysis found decreased ALFF in the right PFCventmed and MFG among individuals with T2DM relative to HCs (54, 55). These contrasting findings indicate that MCI and T2DM differentially affect ALFF in prefrontal regions. ALFF is a stable biomarker of cognitive impairment, and its abnormality in these areas correlates with poorer performance on neuropsychological tests, including the Auditory-Verbal Learning Test and the MoCA (56). Both rs-fMRI and structural MRI studies consistently demonstrate disrupted functional connectivity and spontaneous activity within the DMN in MCI and T2DM, which may contribute to impairments in cognitive ability and episodic memory (57, 58). Furthermore, DMN regions, including the SFG and MFG, show disrupted connectivity with the salience network in MCI (51). Meta-regression analyses further indicate that reduced gray matter volume in the right PFCventmed is associated with lower MoCA scores in T2DM-related cognitive dysfunction (59). Notably, within the T2DM-MCI subgroup, ALFF in the right PFCventmed and MFG correlated positively with 2h-PBG levels, suggesting that functional alterations in these regions may serve as key neurobiological markers of cognitive impairment in T2DM.

### Significant main effect of T2DM on cerebral glucose metabolism and ALFF

Notably, T2DM was identified as the principal factor driving the concurrent hypometabolism and elevated ALFF in the right IFGorb. In T2DM patients, this decoupling likely reflects impaired neuro-metabolic coupling under pathological conditions, potentially originating from central insulin resistance. On one hand, disrupted insulin signaling suppresses the synthesis and release of neurotransmitters in GABAergic neurons, leading to reduced inhibitory tone and a consequent increase in neural excitability, which is manifested as elevated ALFF (46). On the other hand, insulin resistance contributes to reduced cerebrovascular reactivity, impaired glucose transporter function, and reduced mitochondrial energy production efficiency (60–62). Consequently, despite increased neuronal firing, cerebral glucose utilization is impaired, ultimately resulting in the hypometabolism observed on PET. This dissociation between high neural activity and low metabolic efficiency may reflect a state of compensatory yet inefficient hyperactivation in the IFGorb, suggesting its potential role as a core mechanism underlying deficits in executive function and inhibitory control in T2DM.

## Conclusion

Using an integrated PET/fMRI approach, this study observed that T2DM was prominently associated with aberrant cerebral glucose metabolism and ALFF disruption, particularly in the right IFGorb. We also found an interaction whereby T2DM and MCI were linked to opposing ALFF patterns in the right PFCventmed and MFG. Additionally, within the T2DM-MCI subgroup, ALFF in these regions was positively correlated with 2h-PBG levels. These integrated findings suggest a complex interplay between T2DM and MCI at the neural level. The identified frontal lobe alterations might represent potential early indicators for T2DM-related cognitive decline, providing new mechanistic insights and avenues for future research into therapeutic strategies.

## Limitations and future directions

Several limitations of this study warrant consideration. First, the lack of a comprehensive assessment of cerebral small vessel disease (CSVD) (e.g., white matter hyperintensities, microbleeds) represents a methodological limitation. This leaves two critical questions unresolved: whether metabolic dysregulation (e.g., glycemic fluctuations) influences cognitive function independently or via cerebrovascular damage; and whether the observed impairments are specific to T2DM or are confounded by concomitant CSVD, which shares common etiological pathways. Second, although the linear effect of age was controlled through covariate adjustment, the broad age range may still introduce residual heterogeneity not accounted for by the model. Furthermore, the sample size of this study is insufficient to examine potential interactions between T2DM and age. Future studies with larger samples, either stratified by age or focused on specific age groups (such as middle-aged vs. elderly populations), are needed to more precisely elucidate the trajectory of T2DM-related cognitive decline. Third, the high cost of simultaneous PET/fMRI scanning, concerns regarding radiation exposure, and the stringent inclusion and exclusion criteria adopted in this study resulted in a relatively small sample size (n = 54). This may have led to underpowered analyses and could limit the generalizability of the conclusions. Therefore, further validation through larger cohort studies is required to confirm these preliminary findings. Additionally, even with adjustment for MoCA scores and education as a covariate, residual confounding due to between-group differences in educational attainment may not be fully addressed. As education serves as a major proxy for cognitive reserve and may nonlinearly influence brain function, the identified T2DM-MCI-specific neural alterations should be interpreted with caution regarding their specificity as biomarkers. Future studies with longitudinally followed, education-matched cohorts would help to more clearly delineate the specific neuropathological effects of T2DM itself. Finally, establishing causal links between the observed metabolic and ALFF alterations and cognitive decline requires longitudinal data.

## Data Availability

The raw data supporting the conclusions of this article will be made available by the authors, without undue reservation.
